# Challenging risk factors for right and left laparoscopic adrenalectomy: A single centre experience with 272 cases

**DOI:** 10.1590/S1677-5538.IBJU.2019.0131

**Published:** 2019-09-02

**Authors:** Kadir Omur Gunseren, Mehmet Cagatay Cicek, Hakan Vuruskan, Yakup Kordan, Ismet Yavascaoglu

**Affiliations:** 1 Department of Urology, Uludag University, School of Medicine, Nilufer, Bursa, Turkey;; 2 Departmet of Urology, Koc University, School of Medicine, Nilufer, Bursa, Turkey

**Keywords:** Laparoscopy, Adrenalectomy, Risk Factors

## Abstract

**Purpose:**

This study aimed to compare perioperative and postoperative results of right and left laparoscopic adrenalectomy (LA), and to evaluate the impact of challenging factors on these outcomes.

**Materials and Methods:**

A total of 272 patient’s medical records that underwent single side LA between October 2006 and September 2017 were retrospectively reviewed. The patients were divided into 2 groups according to operation side. Moreover, pheochromocytoma, metastatic masses and adrenal lesions >5cm in size were considered to be difficult adrenalectomy cases and the outcomes of these cases were compared between two groups.

**Results:**

135 patients (49.6%) underwent right LA and 137 patients (50.4%) underwent left LA. Operation time, estimated blood loss (EBL) and hospitalization time were similar between the groups (p=0.415, p=0.242, p=0.741, respectively). Although EBL was higher on the right side than the left (p=0.038) in the first 20 cases, after this learning period has been completed, there was no significant difference between the groups. In patients with pheochromocytoma, metastatic mass and a mass >5cm in size, despite bleeding complications were clinically higher on the right side, this difference was not statistically significant.

**Conclusions:**

During the learning period of LA, EBL is higher on the right side. Due to the greater risk of bleeding complications on the right side even on the hands of experienced surgeons, extra care and preoperative planning are required in patients with pheochromocytoma, metastatic masses and masses >5cm in size.

## INTRODUCTION

The laparoscopic treatment of adrenal masses was first described by Gagner et al. in 1992 (1). When compared with open adrenalectomy (OA), laparoscopic adrenalectomy (LA) is known to have better cosmetic results in addition to less blood loss and a shorter length of stay in hospital (2, 3). Despite current controversy in the laparoscopic surgery of large and metastatic adrenal masses, LA has become the standard surgical method for benign adrenal masses (4, 5).

Both of the adrenal glands have different anatomical features. The right adrenal gland has a partial retrocaval localisation, is adjacent to the liver and duodenum and drains to the inferior vena cava (6, 7). It has been reported that LA on the right side could be more difficult because of these differences between the two sides (6, 7). Although there are few studies in literature that have compared adrenalectomy on both sides, different interpretations can be seen in these studies. In this article, considering our large clinical data, we compared the perioperative and postoperative results of right and left LA. Moreover, herein we firstly evaluated the challenging factors, such as surgical experience and histopathological properties on these outcomes.

## MATERIALS AND METHODS

Between 2006 and 2017, data of patients submitted to LA in our clinic were retrospectively reviewed. Patients were excluded if they underwent bilateral LA, had a history of surgery for the same reason, or had a bleeding diathesis or a skeletal deformity. The patients were separated into 2 groups; Group 1 comprised cases submitted to right-side LA, and Group 2 comprised cases submitted to left-side LA.

In accordance with the recommended guidelines, surgery was performed to adrenal lesions expressing hormone with clinical importance, non-functioning lesions that were determined radiologically to have a risk of malignancy and lesions showing a tendency for growth (8, 9). All the operations were performed with a lateral transperitoneal approach method by 3 different surgeons.

The groups were compared in respect of age, gender, mass size, operating time, estimated blood loss (EBL), length of stay in hospital and complications. The measurement in the pathology report was taken as the mass size. The operating time was calculated as the time from entry of the camera and working portals to closure of the incision after removing the mass from the body. EBL was measured by collecting the postoperative drainage amount and subtracting the amount of fluid irrigation from the amount aspirated during the operation.

Based on the previous studies, the first 20 cases in the series were accepted as the learning period and the data of this period were compared (10). In addition, as pheochromocytoma, metastatic masses and adrenal lesions >5cm in size were accepted as difficult adrenalectomy cases, the results of these cases were compared separately for the two groups.

Data obtained in the study were analysed statistically using SPSS v 22 software (Chicago, IL, USA). Categorical data were compared using the Chi-square test. Quantitative data were compared using the Independent Samples t-test and the Mann Whitney U-test. A value of p <0.05 was accepted as statistically significant.

## RESULTS

Totally 272 patients were analysed, comprising 107 (39.3%) males and 165 (60.7%) females. Group 1 included 135 (49.6%) cases of right-side LA, and Group 2 included 137 (50.4%) cases of left-side LA. No statistically significant difference was determined between the groups in terms of age, gender, operating time, EBL, mass size, and length of hospital stay (p=0.888, p=0.130, p=0.415, p=0.242, p=0.184, p=0.741 respectively) (Table-1).

**Table 1:** Comparison of right and left laparoscopic adrenalectomy cases.

	Right Side (n:135)	Left Side (n:137)	P value
**Age**			
Mean (sd)	51±11.7	50.7±11.8	0.888^t^
Median (range)	51 (24-82)	50 (21-88)	
**Gender**			
Male	47 (34.8%)	60 (43.8%)	0.130^ch^
Female	88 (65.2%)	77 (56.2)	
**Operation Time (min)**			
Mean (sd)	98.7±36.6	100.6±33.2	0.415^m^
Median (range)	90 (45-210)	92 (40-210)	
**Estimated Blood Loss (mL)**			
Mean (sd)	59.3±61.2	47±27.5	0.242^m^
Median (Range)	50 (100-500)	50 (10-200)	
**Mass Size (mm)**			
Mean (sd)	41.4±19	39.2±19.3	0.184^m^
Median (range)	40 (10-150)	35 (10-100)	
**Pathology**			
Adenoma	72	68	
Pheochromacytoma	22	19	
Adrenocortical Carcinoma	5	2	
Metastasis	14	20	
Leomyosarcoma	1	0	
Malign Pheochromacytoma	1	0	
Other Benign	20	28	
**Hospital Stay (day)**			
Mean (sd)	2.9±1.5	2.9±2.4	0.741^m^
Median (range)	3 (1-8)	2 (1-26)	

^**t**^ = Independent Simple Test; ^**m**^ = Mann-Withney u Test; **n** = Number; **sd** = Standart Deviation

Considering the first 20 cases as in the learning period, comparisons of the right and left LA of these cases are shown in Table-2. Although the differences were not statistically significant, the mean operating time on the right side was longer than the left side (p=0.766). However, the size of the adrenal mass on both sides was similar, the mean estimated blood loss (EBL) was statistically significantly higher on the right side (55.5±19.2mL) than on the left side (41.1±7.8mL) (p=0.038) (Table-2). After this learning process has been completed, there was no significant difference regarding EBL.

**Table 2:** Comparison of challenging right and left laparoscopic adrenalectomy cases.

	Right Side		Left Side		P value

	Mean ± sd	Min-Max (Median)	Mean ± sd	Min-Max (Median)	
**First 20 Cases**					
**n: (Right:11 / Left:9)**					
Age	52.2±8.3	37-68 (50)	47.7±11	26-64 (48)	0.310^t^
Operation Time (min)	160±36.6	120-210 (180)	154.4±36.1	100-210 (150)	0.766^m^
Estimated Blood Loss (mL)	55.5±19.2	30-100 (50)	41.1±7.8	30-50 (40)	0.038^m^
Mass Size (mm)	33.6±11.6	20-60 (32)	35.6±12.4	20-60 (35)	0.766^m^
Hospital Stay (day)	3.8±1.7	2-7 (3)	3±1	2-5 (3)	0.331^m^
**Pheochromocytoma**					
**n: (Right:22 / Left:19)**					
Age	49.1±14.4	24-75 (50.5)	42.8±13.3	21-65 (43)	0.780^t^
Operation Time (min)	106.6±42.6	45-210 (97.5)	118.7±33.6	45-180 (120)	0.196^m^
Estimated Blood Loss (mL)	75.2±98.4	10-500 (50)	51.1±22.2	25-100 (50)	0.386^m^
Mass Size (mm)	42.6±16.3	10-80 (45)	51.3±16.8	20-80 (52)	0.104^m^
Hospital Stay (day)	3±1.5	1-6 (3)	2.9±1	1-5 (3)	0.871^m^
**Metastasis**					
**n: (Right:14 / Left:19)**					
Age	55.5±8.0	41-71 (54)	58.1±9.2	40-72 (60.5)	0.400^t^
Operation Time (min)	98.5±27.6	60-150 (95)	100±40.1	45-120 (90)	0.823^m^
Estimated Blood Loss (mL)	79.6±75.7	25-300 (50)	47.2±38.8	15-200 (50)	0.148^m^
Mass Size (mm)	38.5±16.7	13-73 (34)	41.3±26.8	10-100 (33.5)	0.877^m^
Hospital Stay (day)	2.6±1.3	1-6 (2.5)	4.7±5.3	1-26 (3)	0.148^m^
**Mass Size >5cm**					
**n: (Right:45 / Left:41)**					
Age	47.1±12.5	24-71 (46)	48.8±12.8	28-88 (46)	0.545^t^
Operation Time (min)	106.2±32.9	60-180 (95)	106.1±31.7	45-180 (100)	0.878^m^
Estimated Blood Loss (mL)	80.6±88.3	10-500 (50)	52.6±40.6	10-200 (50)	0.106^m^
Mass Size (mm)	61.7±18.2	50-150 (55)	62.6±15.7	50-100 (55)	0.606^m^
Hospital Stay (day)	2.8±1.3	1-6 (3)	3.3±3.8	1-26 (3)	0.919^m^

^**t**^ = Independent Simple Test; ^**m**^ = Mann-Whitney u Test; **n** = Number; **sd** = Standard Deviation

The results of the 41 patients submitted to right and left LA because of pheochromocytoma were found to be similar (Table-2). The EBL of the right-side LA was greater than that of the left side, but not to a statistically significant level (75.2±98.4mL vs. 51.1±22.2mL) (p=0.386) (Table-2). In 1 patient (case no: 80) submitted to right LA because of a 5cm right-side adrenal pheochromocytoma, 1 unit erythrocyte (ES) replacement was administered perioperatively because of 500cc blood loss.

No significant difference was determined between the patients submitted to right and left LA because of the pathology report of metastasis (Table-2). The EBL was found to be 79.6±75.7mL in the right side and 47.2±38.8mL in the left-side operations (p=0.148) (Table-2).

The results of the patients submitted to right and left LA because of adrenal mass >5cm were found to be similar (Table-2). The EBL of the right-side LA surgeries for mass >5cm in size was greater than that of the left side but not to a statistically significant level (80.6±88.3mL vs. 52.6±40.6mL) (p=0.106) (Table-2). Open conversion was employed in one patient with diagnosis of 5cm of functional right adrenal adenoma due to excessive bleeding in number of 119th of the series.

## DISCUSSION

With the current prominence of minimally invasive treatments, laparoscopic and robotic methods have started to be used in place of open surgery. Following the first description of minimally invasive adrenalectomy, LA started to be considered as a treatment method (1, 11). Laparoscopy is now applied as the standard in the surgical treatment of several pathologies (6). Although different surgical techniques have been described such as anterior, lateral and retroperitoneal approaches, the lateral transperitoneal approach is the most commonly used (7). The most important advantages of the lateral transperitoneal method are that it provides the opportunity to work in a wider surgical area and there is clearer visualisation of the surrounding organs (12).

The vascular properties and the organs around the adrenal glands, which are located in the upper retroperitoneal area, are different on the right and left sides. Due to these anatomic differences between the right and left adrenal glands, there is an opinion that right-side LA is more difficult than left-side (6, 7, 13). Moreover, there are different interpretation in studies in literature that have compared the perioperative data of right and left LA. Although right-side surgery has been reported as shorter in some series made with an anterior approach there are also studies that have emphasised that the operating time is similar on both sides (14, 15). In cases submitted to the transperitoneal method, Reider et al. reported that right-side surgery was shorter and tended to be less bleeding on the right side (6). Although Cianci et al. found the operating time to be longer on the left side in cases submitted to the lateral transperitoneal method, it was emphasised that left side surgery was more complex (7). The longer operating time on the left side was associated by both of these authors with renal hilus dissection and mobilisation of the spleen and splenic colonic flexure during adrenalectomy. In the current study, the operating times and blood loss values were found to be similar on both sides (Table-1). This shows that when the surgeon is sufficiently experienced, renal hilus dissection and mobilisation of the spleen and splenic colonic flexure, which are thought to make left-side surgery more complex, can be applied quickly and without problems.

Although there are different views in literature related to the LA learning curve, Higashihara et al. reported this period to be 20 cases (10, 16-19). In the first 20 cases of the current series, despite larger-sized left-side masses, the amount of bleeding was greater in right-side operations, but this difference was eradicated as the number of cases increased (Table-2). This shows that in adrenalectomies performed before sufficient experience is acquired, there is a greater possibility of bleeding from the right side, even if the mass is of small dimensions, and when a certain level of experience is reached, the bleeding risk on the right side was seen to decrease.

Even for experienced surgeons, laparoscopic surgery of pheochromocytoma has been reported to be more difficult than other adrenal lesions (20). In addition, surgery of metastatic lesions may be more difficult because of adhesions to surrounding tissues, and masses >5cm have been reported to constitute a risk for conversion of laparoscopy to open surgery (21). Despite the similarity of preoperative results and length of hospital stay of the adrenalectomy cases in the current study with this difficult adrenalectomy cases, it was noticeable that the mean amount of bleeding was greater from the right side than from the left in all three groups (Table-2). Furthermore, as the cases with bleeding requiring ES replacement and the one case that was converted to open surgery were on the right side, this showed that right-side LA could be more dangerous than left-side LA in difficult adrenalectomy cases. However, experienced the surgeon is, if surgery is performed because of a pheochromocytoma, metastasis or a mass >5cm, on the right side, more care must be taken. It is also important that in these difficult cases where there is the possibility of sudden bleeding, the anaesthetist must be warned for a broad vascular route or central catheterisation to be able to make the necessary rapid intervention and the operation should only be started after the preoperative preparation of ES.

With the exception of patients submitted to bilateral adrenalectomy, all patients submitted to LA were included in the study. Therefore, there was no selection bias in this study. However, that the study was retrospective and that the operations were performed by 3 different surgeons, albeit of similar experience, can be considered limitations of the study.

## CONCLUSIONS

In conclusion, although the adrenal glands show different anatomic features on the right and left sides, after acquiring a certain level of experience, the results of right and left LA are similar and the operations have an equal degree of difficulty. However, as there is a greater risk of bleeding during right-side LA performed in the learning period, right-side LA can be said to be more difficult for a surgeon at this stage. Furthermore, even for experienced surgeons, as there is a greater risk of bleeding on the right side, extra care and preoperative planning are required for pheochromocytoma, metastatic masses and masses >5cm in size.
